# Drug Allergy Pharmacogenetics: A Clinical Perspective

**DOI:** 10.1007/s11882-026-01307-z

**Published:** 2026-11-12

**Authors:** Rishitha Nalabothu, Matthew S. Krantz, Santiago Alvarez Arango, Eric M. Mukherjee, Elizabeth J. Phillips

**Affiliations:** 1 Vanderbilt University School of Medicine, Nashville, TN USA; 2 Division of Allergy and Immunology, Departments of Medicine and Pediatrics, UT Southwestern Medical Center, Dallas, TX USA; 3 Center for Drug Safety and Immunology, Department of Medicine, Vanderbilt University Medical Center, Nashville, TN USA; 4 Vanderbilt University Medical Center, 1161 21st Avenue South, Nashville, TN A-2200 MCN, 37232 USA

**Keywords:** Severe cutaneous adverse reactions, HLA, Stevens–Johnson syndrome, Drug hypersensitivity, Pharmacogenetics, Immune-mediated drug reactions

## Abstract

**Purpose of review:**

Genetic variation contributes both to dose-related adverse drug effects and to immune-mediated drug reactions, but these two forms of risk sit in different parts of the genome. We summarize what the practicing allergist and immunologist needs to know about pharmacogenetics, with emphasis on human leukocyte antigen (HLA)-associated delayed reactions, and propose a framework for combining genotype with phenotype, drug chronology and established allergy testing.

**Recent findings:**

A small number of drug–HLA pairs are clinically actionable, and pre-prescription screening has reduced abacavir hypersensitivity, carbamazepine-induced Stevens-Johnson syndrome and toxic epidermal necrolysis, and allopurinol-induced severe cutaneous adverse reactions in targeted populations. Newer associations show that single-allele screening leaves at-risk patients unidentified and that the dominant risk allele is itself population-dependent.

**Summary:**

Genotype is best used as a risk modifier and diagnostic adjunct rather than a stand-alone test. Incomplete positive predictive value, residual risk after a negative result, and underrepresentation of geographically heterogeneous populations in discovery cohorts remain the principal barriers to wider implementation.

## Introduction

Pharmacogenetics refers to the effect of variation in a single gene on drug response, whereas pharmacogenomics considers the contribution of variation across the genome [1, 2]. The most clinically actionable examples in allergy and immunology are associations between human leukocyte antigen (HLA) alleles and delayed, T-cell–mediated drug reactions, which have enabled pre-prescription screening, informed safer drug selection, and clarified immunologic mechanism [1, 2].

**Fig. 1:**
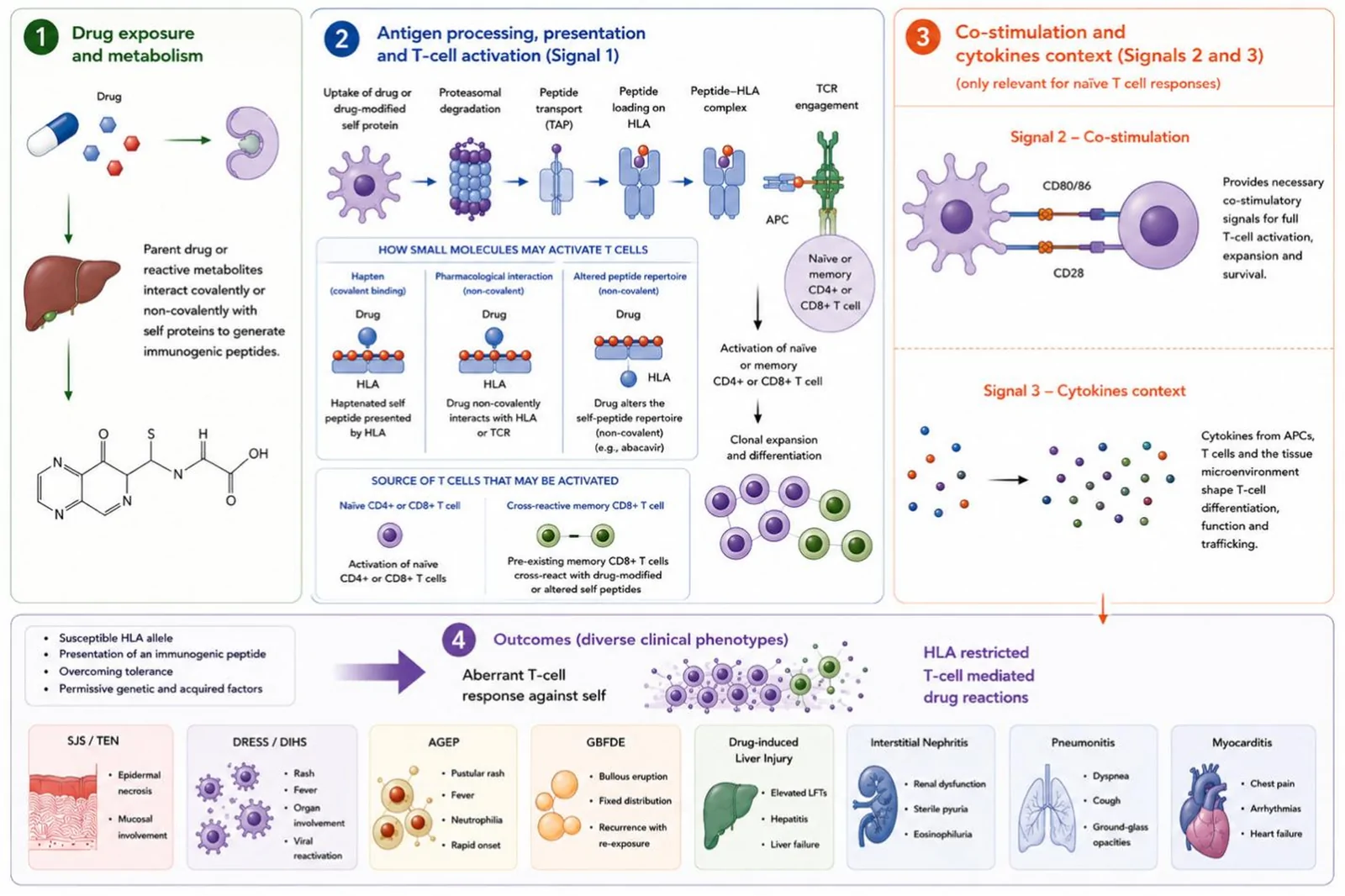
Immunopathogenesis and risk of HLA-restricted T-cell–mediated drug reactions. Panel 1: parent drug or reactive metabolites interact covalently or non-covalently with self proteins to generate immunogenic peptides. Panel 2: uptake, proteasomal degradation, TAP-mediated peptide transport and loading onto HLA produce the peptide–HLA complex that engages the T-cell receptor (signal 1); the inset summarizes the three non–mutually exclusive models by which small molecules activate T cells (hapten formation, pharmacological interaction with immune receptors, and alteration of the self-peptide repertoire) and the two sources of responding cells (naïve CD4+ or CD8+ T cells and cross-reactive memory CD8+ T cells). Panel 3: co-stimulation through CD80/86–CD28 (signal 2) and the cytokine context (signal 3) determine differentiation, function and trafficking, and are principally relevant to naïve responses. Panel 4: the resulting phenotypes, spanning SJS/TEN, DRESS/DIHS, AGEP and GBFDE as well as organ-specific reactions including drug-induced liver injury, interstitial nephritis, pneumonitis and myocarditis. Under the 2026 EAACI nomenclature the hapten and altered peptide repertoire models correspond to drug-antigen-driven reactions (drug allergy), whereas pharmacological interaction corresponds to drug-directly-driven reactions (drug hypersensitivity)

Adverse drug reactions were historically divided into Type A, predictable and dose-dependent and explicable by the drug’s pharmacology, and Type B, unpredictable, dose-independent and frequently immunologic [2, 3]. The severe cutaneous adverse reactions (SCARs), comprising Stevens-Johnson syndrome and toxic epidermal necrolysis (SJS/TEN), drug reaction with eosinophilia and systemic symptoms (DRESS), acute generalized exanthematous pustulosis (AGEP), and generalized bullous fixed drug eruption (GBFDE), sit within the Type B group (Fig. 1). Although individually rare, they carry high mortality, frequent chronic ocular, cutaneous and respiratory sequelae, and hospitalization costs comparable to burns of similar body surface involvement, which is what makes prevention through screening a high-value intervention [2, 3].

The 2026 EAACI position paper replaces this binary definition with a nomenclature built on three elements: chrono-morphologic phenotype, mechanistic endotype, and the characteristics of the drug [4]. Immediate drug reactions occur up to 6 h after dosing; delayed reactions occur beyond 6 h and span delayed exanthematous eruptions, SJS/TEN, DRESS, AGEP, GBFDE, serum sickness-like reactions, cytopenias, and organ-specific reactions including drug-induced liver injury (DILI). Endotype separates drug-antigen-driven reactions, in which the drug modifies host protein to generate an antigen and sensitization can be demonstrated by skin or in vitro testing, from drug-directly-driven reactions, in which the drug engages immune receptors or inflammatory pathways non-covalently and reversibly without forming an antigen. Under this nomenclature drug allergy denotes the former and drug hypersensitivity the latter, and the terms are no longer interchangeable [4]. Two consequences matter here. Phenotype is not pathognomonic of endotype, and the same delayed phenotype can arise from either. Confirmatory testing for the drug-directly-driven endotype is largely unavailable, so involvement is inferred by exclusion, leaving genotype as one of the few objective data points in that space [4].

This review summarizes the pharmacogenomics of immune-mediated drug reactions with emphasis on HLA-associated delayed reactions, and considers two clinical uses of genetic information: prospective risk prediction before exposure, and post-reaction attribution among multiple potential culprit drugs.

## Where Genetic Risk Resides in Adverse Drug Reactions

### Dose-related and Off-target Effects

Hereditary risk depends on the type of reaction being evaluated. Variation underlying dose-dependent pharmacologic and off-target effects clusters in genes governing pharmacokinetics, including metabolic enzymes and transporters, as with CYP2C9 and VKORC1 and warfarin response, CYP2D6 ultrarapid metabolism and codeine, SLCO1B1 and statin-associated muscle symptoms, and SLC22A2 and cisplatin nephrotoxicity. Variation underlying immunologically mediated reactions localizes to the MHC, so that most clinically relevant drug allergy associations are effectively single-locus HLA pharmacogenetics [1, 4, 5].

### Immediate Reactions

Immediate reactions, including urticaria, angioedema and anaphylaxis, arise from drug-specific IgE or from direct, non-IgE mast-cell activation through receptors including MRGPRX2, as with fluoroquinolones, neuromuscular blocking agents and opioids [6]. Under the EAACI nomenclature IgE-mediated reactions are drug-antigen-driven, whereas MRGPRX2-mediated activation, direct complement activation, COX-1 inhibition–mediated reactions and cytokine release reactions are drug-directly-driven [4]. None of these endotypes currently has a validated predictive genetic test.

MRGPRX2 is the best-studied candidate gene. Naturally occurring missense variants are functionally consequential: several in the extracellular and transmembrane domains cause loss of function, failing to degranulate in response to substance P, hemokinin-1, human β-defensin-3 or icatibant, while variants in the transmembrane and intracellular domains can confer gain of function [7, 8]. Variants identified in patients reacting to quinolones and vancomycin show amplified, drug-specific activation in vitro [9]. Individual variants are rare, the direction of effect differs between them, and no prospective study has linked genotype to reaction risk at the bedside.

The clearest genetic association with an immediate reaction is class II rather than mast-cell related. Asparaginase hypersensitivity, including anaphylaxis, in children treated for acute lymphoblastic leukemia is associated with HLA-DRB1*07:01 and is greatest in carriers of the extended HLA-DRB1*07:01–DQA1*02:01–DQB1*02:02 haplotype [10, 11]. The reaction is antibody-mediated, yet genetic control sits at the class II–restricted step of CD4 + T-cell help for anti-asparaginase antibody production. Asparaginase is also a bacterial protein rather than a small molecule, a reminder that the pharmacogenetics of biologicals need not resemble that of small molecules.

Associations have also been reported with beta-lactams, cephalosporins and iodinated radiocontrast media, together with non-MHC variants in genes governing IgE responses and mediator release [12–15]. Two features separate these from the class I associations that dominate delayed reactions. Effect sizes are far smaller, with odds ratios typically below two against values in the tens to hundreds for HLA-B*57:01 and abacavir. And the phenotype is often imprecise. A genome-wide analysis of self-reported penicillin allergy across three biobanks and more than 600,000 individuals identified HLA-B*55:01 with an odds ratio of approximately 1.4, replicated in an independent cohort of over one million participants [16]. The association is robust and reproduced, but an electronic health record penicillin allergy label mixes immediate reactions, delayed exanthems, intolerances and mislabels, so until the phenotype is resolved such signals are of mechanistic rather than clinical interest.

### Delayed Reactions

Most immune-mediated drug reactions are benign cutaneous eruptions that are likely T-cell mediated but are often not reproducible on drug rechallenge and generally lack strong consistently replicated HLA or other genetic associations. This contrasts with severe immune-mediated adverse drug reactions, including severe cutaneous adverse reactions (SCAR) such as Stevens-Johnson syndrome and toxic epidermal necrolysis (SJS/TEN) and drug reaction with eosinophilia and systemic symptoms (DRESS) as well as drug-induced liver injury (DILI). These severe phenotypes are characterized by robust, drug-specific memory T-cell responses and for many drug-phenotype combinations strong genetic associations, particularly with HLA class I [1, 4]. HLA molecules shape the repertoire of drug-associated peptides presented to T cells, with class I HLA-restricted responses activating drug-specific CD8 + T cells that are particularly important in many severe reactions. Although non-HLA genetic factors can also modify susceptibility, HLA associations generally represent the strongest and most reproducible genetic risk factors identified to-date. Consequently, pharmacogenetic screening has been most clinically actionable for severe, well-defined immune-mediated phenotypes with strong drug-HLA associations and underpins several successfully implemented screening programs. Subsequent sections address the clinical phenotypes and immunologic mechanisms underlying these reactions and specific drug-HLA associations.

## Clinical Phenotypes of Delayed Drug Reactions

Accurate phenotyping of SCAR, including the pattern and severity of associated organ involvement, is fundamental to epidemiologic and genetic association studies. Misclassification or aggregation of clinically and biologically distinct phenotypes can dilute or obscure true genetic associations. Aside from being essential for good acute and follow-up clinical care, rigorous, standardized phenotyping is therefore essential to define reproducible genotype-phenotype relationships and identify clinically meaningful genetic risk factors.

### The SCAR Phenotypes and their Mimics

The SCAR phenotypes share immune-mediated keratinocyte damage but differ in distribution, intensity and timing (Fig. 1). SJS and TEN lie on a continuum defined by the extent of epidermal detachment, below 10% for SJS, 10–30% for overlap, and above 30% for TEN respectively, and typically develop 4–28 days after exposure with a febrile prodrome followed by mucosal involvement, painful targetoid or atypical macules and sloughing with a positive Nikolsky sign [17]. DRESS occurs 2–8 weeks after exposure with extensive exanthem, facial edema, lymphadenopathy, eosinophilia, atypical lymphocytosis, organ involvement and reactivation of HHV-6, EBV or CMV [18]. AGEP appears within days as multiple small sterile pustules on an erythematous base with fever and neutrophilia, resolving without sequelae after drug cessation. GBFDE recurs at fixed anatomic sites, with rapid blistering over a significant body surface on re-exposure while sparing previously unaffected skin [19].

Recognizing mimics matters because early withdrawal of the culprit improves outcome. Erythema multiforme major (EM) can be distinguished from SJS/TEN by lesion morphology and distribution, the degree of epidermal detachment, and its usual trigger. EM is characterized by raised, target-like lesions distributed symmetrically on acral/extensor surfaces whereas SJS/TEN presents with flat, atypical targets and dusky purpuric macules that coalesce, favoring the face and trunk. Epidermal detachment is minimal in EM but progresses to confluent blistering and sloughing in SJS/TEN. EM most often has an infectious trigger such as herpes simplex virus or Mycoplasma pneumoniae whereas SJS/TEN is typically drug-induced [17]. Delayed exanthematous eruptions, previously termed maculopapular exanthema, lack the systemic features of DRESS and the epidermal detachment of SJS/TEN. Staphylococcal scalded skin syndrome causes superficial peeling without mucosal involvement, predominantly in young children. Drug-induced hypersensitivity syndrome, as defined in Japan, requires demonstrated HHV-6 reactivation whereas RegiSCAR criteria treat reactivation as supportive, a distinction that matters because drug-induced hypersensitivity syndrome carries a more protracted course and higher risk of late autoimmune sequelae such as thyroiditis and type 1 diabetes [2, 18]. GBFDE and SJS/TEN share full-thickness epidermal necrosis histologically but differ by a deeper dermal lymphocytic infiltrate with eosinophils, recurrence at fixed sites and absence of a systemic prodrome [19]. Non-drug induced mimics include TEN-like acute cutaneous lupus in the acute syndrome of apoptotic pan-epidermolysis (ASAP) spectrum, which presents with a photodistributed eruption, subacute progression over days to weeks, ANA/SSA-Ro/dsDNA positivity, and dermal mucin on histology. There is no established single HLA molecule that restricts the pathogenic cytotoxic response in TEN-like lupus analogous to drug-specific SJS/TEN. TEN-like bullous pemphigoid can mimic TEN when an aged blister roof necroses and is distinguished by tense bullae, negative Nikolsky sign, eosinophil-rich infiltrate, and linear IgG/C3 at the basement membrane on direct immunofluorescence. Acute graft-versus-host disease can produce clinically and histologically indistinguishable TEN-like skin findings in the transplant setting [3].

### Epidemiology and Severity Assessment

**Fig. 2:**
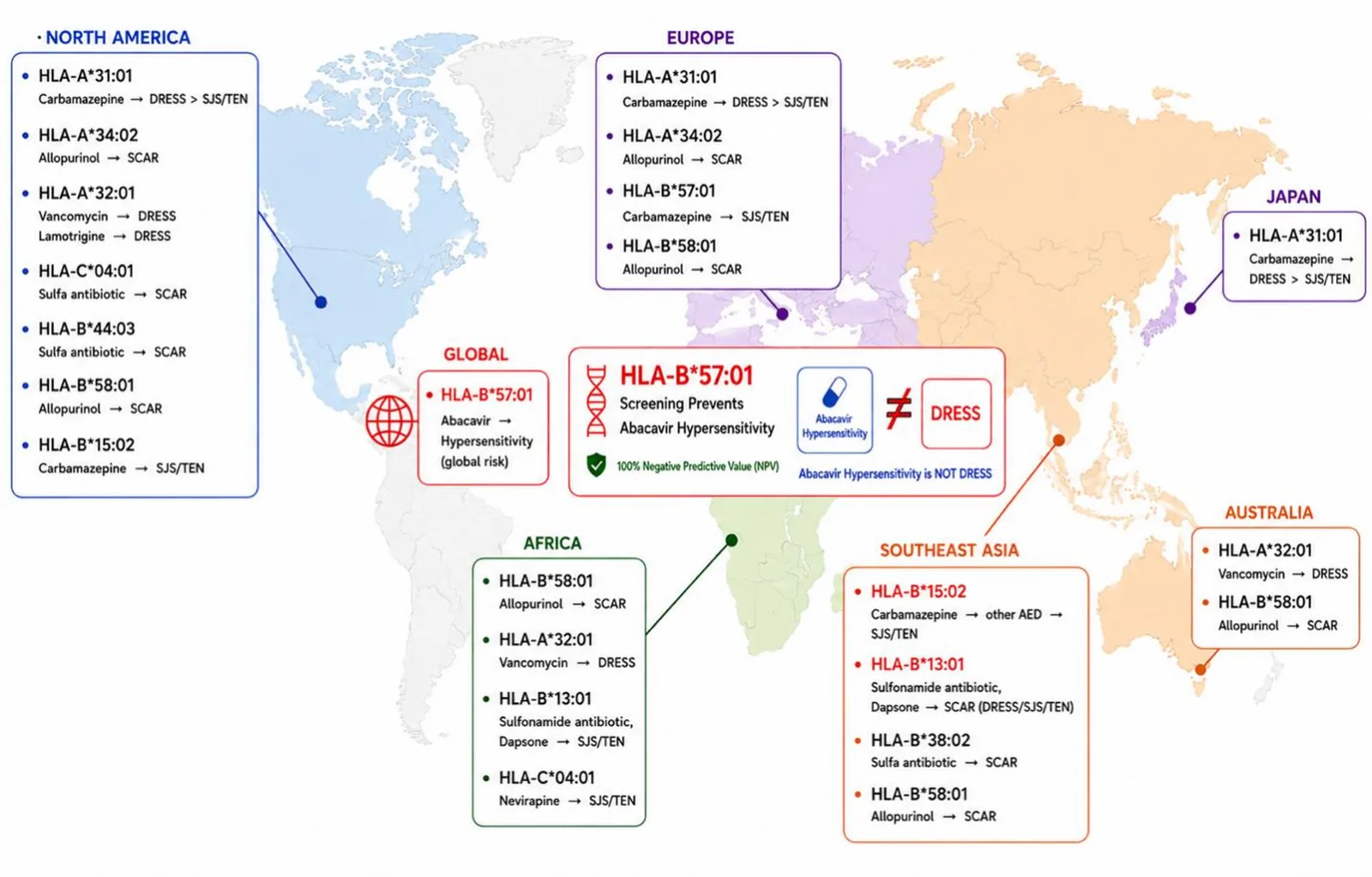
Key HLA risk alleles for T-cell–mediated drug reactions by geographic region. Abacavir and HLA-B*57:01 is the only association that applies globally with 100% negative predictive value; abacavir hypersensitivity is a distinct clinical entity and is not DRESS. Every other association is population-dependent. HLA-B*58:01 confers allopurinol risk across Asian, African, European, North American and Australian populations, whereas the alleles conferring risk of sulfonamide-associated SCAR differ by region: HLA-B*13:01 and HLA-B*38:02 predominate in Southeast Asia, while HLA-B*44:03, HLA-B*38:01 and HLA-C*04:01 account for most risk in the United States and South Africa despite sharing peptide-binding specificities with the Southeast Asian alleles. Carbamazepine illustrates the same principle within one drug, with HLA-B*15:02 predicting SJS/TEN in Southeast Asian populations and HLA-A*31:01 predicting DRESS more than SJS/TEN in European, North American and Japanese populations

SCAR incidence varies geographically, driven mainly by differences in drug utilization and by the frequency of HLA risk alleles (Fig. 2) [3, 20]. Rates of SJS/TEN are below 10 per million person-years in the United States and are higher in African and Asian populations, and immunosuppression, HIV infection, malignancy and chronic kidney disease increase risk [20, 21]. SCORTEN predicts SJS/TEN mortality from seven variables collected within 24 h of admission, although its calibration under modern intensive supportive care has been questioned; ABCD-10 uses five readily available clinical variable to predict SJS/TEN mortality. Although performance was comparable to SCORTEN in the derivation cohort, external validation has shown variable calibration, including mortality underestimation in some cohorts without consistent superiority over SCORTEN [22–25]. RegiSCAR criteria score DRESS on seven parameters [18]. The Drug Hypersensitivity Quality of Life questionnaire addresses subjective burden, though neither captures the long-term ocular, cutaneous and pulmonary consequences experienced by survivors [26].

## Immunologic Mechanisms of HLA-Associated Immune-Mediated Drug Reactions

### Structure, Function and Polymorphism of HLA

Endoplasmic reticulum aminopeptidases (ERAP1 and ERAP 2) trim precursor peptides prior to HLA loading and shape the presented repertoire. Therefore, ERAP polymorphisms modify HLA-restricted immunity, a mechanism that is relevant to the abacavir hypersensitivity described later [27]. Class I molecules encoded by HLA-A, HLA-B and HLA-C are expressed on nearly all nucleated cells and present peptides of 8–10 residues to CD8 + T cells. The binding cleft is formed by the α1 and α2 domains with a floor of β-pleated sheet, and walls formed by their α-helices, and contains six pockets (A–F), of which the F pocket has emerged as a principal site of drug interaction. Class II molecules (HLA-DR, -DQ, -DP) are expressed on professional antigen-presenting cells and present longer exogenous peptides of 13–18 residues to CD4 + T cells as open-ended grooves permit overhang. While class I is more associated with drug reactions, class II variation is more classically associated with autoimmune disease, although exceptions exist in both classes [30, 31]. The HLA locus is the most polymorphic region of the human genome, with more than 43,000 alleles now cataloged, and that polymorphism is the primary heritable determinant of delayed drug reactions and of ancestry-related differences in risk [28–31].

### From Drug Recognition to T-cell Activation

T-cell activation in these reactions follows the same three-signal architecture as any adaptive immune response, and Fig. 1 is organized around it. Signal 1 is delivered when a peptide–HLA complex engages the T-cell receptor; what is distinctive about drug reactions is only how that complex acquires a drug-related determinant. In the hapten/pro-hapten model the drug or a reactive metabolite binds covalently to self-protein, which is then processed and presented as a genuinely novel peptide, the best-supported explanation for beta-lactam and flucloxacillin reactions [32, 33]. In the pharmacological interaction (p-i) model the drug binds non-covalently and reversibly to HLA or to the T-cell receptor without antigen processing, as proposed for sulfamethoxazole and oxypurinol [34]. In the altered peptide repertoire model non-covalent occupancy of the cleft, classically the F pocket of HLA-B*57:01 by abacavir, changes which self-peptides are presented and read as foreign [35, 36]. Hapten and altered peptide repertoire mechanisms generate an antigen and are therefore drug-antigen-driven, whereas p-i is drug-directly-driven [4].

Whichever mechanism creates the ligand, most of what determines whether a clinical reaction follows happens downstream of it. Signal 1 alone is insufficient for a naïve T cell: co-stimulation through CD80/CD86 engagement of CD28 (signal 2) is required for full activation, clonal expansion and survival, and in its absence anergy or deletion is the likely outcome. The cytokine context supplied by antigen-presenting cells and the tissue microenvironment (signal 3) then shapes differentiation, effector function and trafficking, and does much to determine which phenotype emerges from an identical initiating event. Pre-existing cross-reactive memory CD8 + T cells, primed against unrelated and often viral antigens, have lower activation thresholds and reduced dependence on co-stimulation, which is one explanation for reactions occurring on first documented exposure. Carriage of a risk allele determines only whether signal 1 can be delivered; it says nothing about whether co-stimulation is available, what cytokine context prevails, whether cross-reactive memory exists, or whether regulatory mechanisms will contain the response. That is why the same allele and the same drug can produce SJS/TEN in one patient, DRESS in another and no reaction at all in the great majority. Immune checkpoint inhibitors make the same point pharmacologically, lowering the threshold for activation of pre-existing drug-specific, cross-reactive or autoreactive T cells without themselves binding HLA [3].

### Effector Profiles and Emerging Mechanisms

Effector subsets differ by phenotype (Fig. 1). SJS/TEN is driven by resident effector memory CD8 + cytotoxic T cells producing TNF, perforin, granzyme B and granulysin at the zdermal epidermal junction keratinocyte interface, with IL-15 correlating independently with severity and mortality and massive clonal expansions of polyfunctional CD8 + cells in skin and blood, including drug-specific public T-cell receptors [37–40]. DRESS shows a type 2 immune polarization with IL-4, IL-5 and IL-13, and eosinophil recruitment, Sequential herpesvirus reactivation, particularly HHV-6 occurs in a subset of patients and may amplify immune activation and contribute to its protracted, relapsing course. Single-cell transcriptomics in a single-patient report with refractory DRESS showed over-expression of JAK/STAT pathway genes and this patient did response to the JAK 1/3 inhibitor tofacitinib [41]. More recent multi-patient endotyping and clinical studies show that DRESS is immunologically heterogeneous rather than uniformly Th2/Tc2, with mixed type-2, interferon, and other inflammatory endotypes [42, 43]. AGEP involves Th17/Tc17 responses producing IL-8, IL-17, IL-22, IL-36 and GM-CSF, recruiting neutrophils into sterile pustules. GBFDE shares effector molecules with SJS/TEN but is mediated by resident memory CD8 + T cells at prior exposure sites. Spatial proteomics in TEN skin has shown JAK/STAT activation with a type I and type II interferon signature, raising interest in JAK inhibitors [44–46]. Neutrophil extracellular traps induced by lipocalin-2 released from activated CD8 + cells contribute to keratinocyte necroptosis [47]. None of these has entered routine practice, but each is being evaluated as a biomarker of susceptibility or treatment response.

## Actionable Gene–Drug Associations

Over the last two decades, drug–HLA associations and their markedly uneven distribution across populations (Fig. 1) have changed how SCAR is predicted and prevented. Test performance and guideline status for the actionable pairs are summarized in Table 1.

### Abacavir: A Completed Translational arc

Abacavir remains the only drug–HLA association to have completed the full translational path, and it defines what success requires. At T1, HLA-B*57:01 carriage was reported in 2002 to be strongly associated with abacavir hypersensitivity [48]. Crystallographic and immunopeptidomic work then showed that abacavir occupies the F pocket of HLA-B*57:01 and alters the presented self-peptide repertoire [35, 36]. At T2, the PREDICT-1 randomized trial eliminated immunologically confirmed hypersensitivity through prospective screening [49]. The SHAPE study established a negative predictive value approaching 100% in both White and Black patients [50]. At T3, regulatory labeling changed and CPIC recommendations made screening a prerequisite to prescribing [51]. At T4, screening is standard of care, immunologically confirmed hypersensitivity has become rare, and the strategy is cost-effective. Four features explain why abacavir translated: a single allele accounts for essentially all risk, the negative predictive value is near-complete across ancestries, the decision is binary and prospective, and effective alternatives exist. Where any of these fails, as for carbamazepine, allopurinol and co-trimoxazole, the same screening logic does not transfer intact.

### Established Associations

Chung and colleagues reported in 2004 that HLA-B*15:02 was strongly associated with carbamazepine-induced SJS/TEN in Southeast Asian populations [52]. Taiwan introduced nationwide pre-prescription screening in 2010, and CPIC advises avoidance in treatment-naive carriers regardless of ancestry. Hung and colleagues identified HLA-B*58:01 as a marker for allopurinol SCAR in 2005, now screened nationally in Taiwan and Thailand [53]. Konvinse and colleagues identified HLA-A*32:01 as the major determinant of vancomycin DRESS, with risk concentrated in prolonged courses [54]. Together these illustrate when pharmacogenetic testing is clinically useful: a well-established drug–allele relationship, a severe phenotype, and an acceptable therapeutic alternative. Additional foundational associations include HLA-A*31:01 for carbamazepine-induced DRESS in European, Japanese and Korean populations, HLA-B*57:01 for carbamazepine SJS/TEN in European patients, and HLA-B*13:01 for dapsone hypersensitivity syndrome in Chinese populations [55–57].

### Emerging and Population-specific Associations

Co-trimoxazole illustrates the problem of transferability most sharply. HLA-B*13:01 and HLA-B*38:02, initially identified as risk alleles in Southeast Asian populations, were uncommon or absent in adjudicated cohorts from the United States and South Africa. Instead HLA-B*44:03, HLA-B*38:01 and HLA-C*04:01 were independently associated with SJS/TEN in the United States, while HLA-B*44:03 was associated with DRESS in South Africa; HLA-B*13:01 was present in only one of 63 US cases [58]. Importantly these population-specific alleles were not functionally unrelated: HLA-B*44:03 shares peptide-binding specificity with HLA-B*13:01 and HLA-B*38:01 with HLA-B*38:02. Together with HLA-C*04:01, these share-specificity groups identified 94% of US SJS/TEN and 78% of US DRESS cases. Molecular docking further predicted preferential interaction of sulfamethoxazole and its reactive metabolite over trimethoprim with implicated HLA molecules [58]. These findings suggest that shared structural and peptide-binding properties of the HLA binding cleft, rather than individual alleles may provide a more transferable framework for understanding and unifying genetic and biological risk across populations.

Allopurinol makes a related point within a single country. In a United States cohort of adjudicated allopurinol-induced SCAR, HLA-B*58:01 was reproduced in a predominantly self-identified Black population, carried by 62.5% of cases against 5.6% of allopurinol-tolerant matched controls, with homozygosity conferring further risk [59]. More consequentially, the allele was absent in over a third of cases, and HLA-A*34:02, previously linked to allopurinol-associated liver injury, emerged as an independent risk factor, the two alleles together accounting for over 80% of cases [59]. The closely related HLA-B*58:02 would not be detected by HLA-B*58:01-specific assays at all. The message for practice is consistent: which allele carries the dominant risk is a property of the population, not of the drug alone, so a negative single-allele result is considerably less reassuring outside the population in which that allele was discovered. HLA-A*32:01 has recently been associated with lamotrigine-induced DRESS in a US case-control cohort, representing the first HLA allele to be strongly associated with lamotrigine DRESS and extending an allele previously linked to vancomycin [60].

### Drug-induced Liver Injury

HLA associations with DILI are well characterized, are frequently class II, and often differ from those for SCAR caused by the same drug [61, 62]. Flucloxacillin-associated liver injury is associated with HLA-B*57:01, the same allele as abacavir hypersensitivity but with an entirely different phenotype and a number needed to test that is orders of magnitude higher. Amoxicillin-clavulanate liver injury is associated with the HLA-DRB1*15:01–DQB1*06:02 haplotype, lapatinib with HLA-DQA1*02:01, minocycline with HLA-B*35:02 and terbinafine with HLA-A*33:01 in European ancestry patients with the closely related HLA-A*33:03 allele being implicated in East Asian patients. Allopurinol and carbamazepine show divergence within a single drug, HLA-A*34:02 and HLA-A*31:01 respectively being associated with liver injury as well as with cutaneous phenotypes. Because DRESS itself frequently involves the liver, the boundary between DRESS and DILI is not absolute, and Table 1 notes where a single allele is associated with both. These associations are almost entirely diagnostic rather than preventive, since the low incidence of DILI makes prospective screening impractical for every agent studied so far [62].

**Table 1:** Test performance and guideline status of reported drug–HLA associations across SJS/TEN, DRESS and drug-induced liver injury

Drug	Risk allele	Phenotype	Population(s) validated	NNT to prevent one case	Guideline / regulatory status
Abacavir	HLA-B*57:01 [48–50]	Abacavir hypersensitivity syndrome (not DRESS); same allele causes flucloxacillin DILI	All ancestries; NPV validated in White and Black patients	13	CPIC; FDA boxed warning; screening mandatory before prescribing
Allopurinol	HLA-B*58:01 [53]	SJS/TEN, DRESS; also DILI	Han Chinese, Thai, Korean; US Black; European; Australian	250	CPIC; ACR conditional recommendation in high-prevalence groups
Allopurinol	HLA-A*34:02 [59]	SJS/TEN, DRESS; also DILI	US (admixed, predominantly Black), European (Portuguese)	Not established	Not in guidelines; independent additional risk factor
Amoxicillin-clavulanate	HLA-DRB1*15:01–DQB1*06:02 [62]	DILI (cholestatic/mixed)	European	Not established	Not in guidelines; diagnostic use only
Carbamazepine	HLA-B*15:02 [52]	SJS/TEN	Southeast Asian, Han Chinese, Thai	1000	CPIC; FDA label; screening mandated in Taiwan, Thailand, Singapore, Hong Kong
Carbamazepine	HLA-A*31:01 [55]	DRESS > SJS/TEN; also DILI	European, North American, Japanese	5000	CPIC; recommended where alternatives exist; not routinely mandated
Co-trimoxazole	HLA-B*13:01, B*38:02 (SE Asia); B*44:03, B*38:01, C*04:01 (US, South Africa) [58]	SJS/TEN, DRESS; DILI also reported	Southeast Asian; US; South African	211 to > 1000	Not in guidelines; alleles differ by region but share peptide-binding specificity
Dapsone	HLA-B*13:01 [57]	DRESS (hepatic involvement common)	Chinese, Thai, Indonesian leprosy programs	84	Screening implemented in leprosy control programs
Flucloxacillin	HLA-B*57:01 [61]	DILI (cholestatic)	European	> 10,000	Not in guidelines; same allele as abacavir, different phenotype
Lamotrigine	HLA-A*32:01 [60]	DRESS	European	2500	Not in guidelines
Lapatinib	HLA-DQA1*02:01 [62]	DILI (hepatocellular)	European; multi-ethnic trials	Not established	Referenced in product labeling; not mandated
Minocycline	HLA-B*35:02 [62]	DILI (autoimmune-like); minocycline also causes DRESS	European, US	Not established	Not in guidelines; diagnostic use only
Nevirapine	HLA-C*04:01 (with CYP2B6 slow metabolizer) [69, 70]	SJS/TEN	Malawian, Mozambican, sub-Saharan African	200	Not in guidelines; nevirapine now rarely used
Phenytoin	HLA-B*15:02 plus CYP2C9*3 [68]	SJS/TEN, DRESS; DILI also reported	Southeast Asian (HLA); multiple (CYP2C9)	Not established	CPIC; dose reduction advised in CYP2C9 intermediate and poor metabolizers
Terbinafine	HLA-A*33:01 [62]	DILI (cholestatic)	European	Not established	Not in guidelines; diagnostic use only
Vancomycin	HLA-A*32:01 [54, 71]	DRESS	Multi-ethnic US; prolonged therapy	75	Not in guidelines; supports attribution after a reaction

**Fig. 3:**
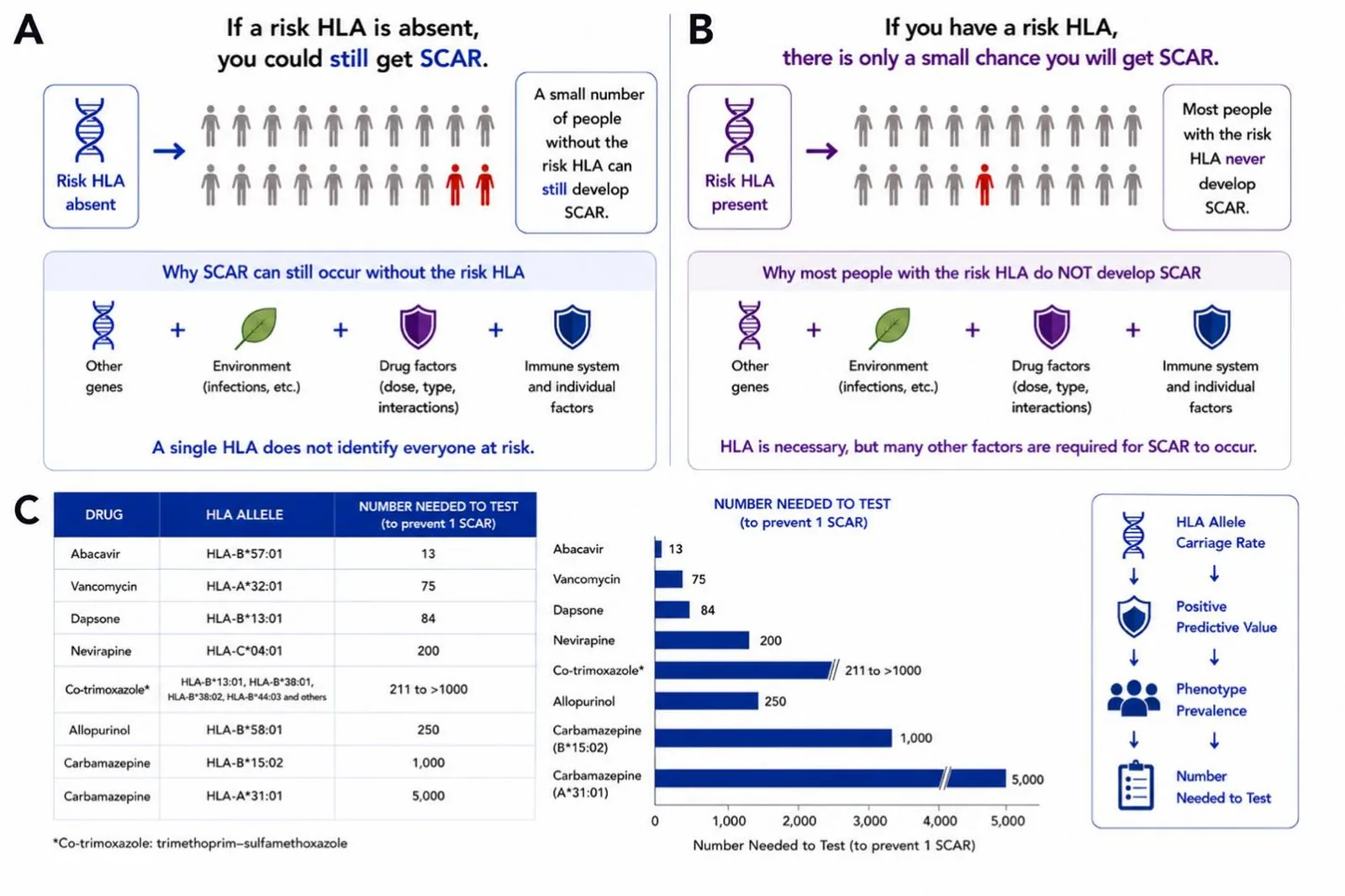
HLA carriage is necessary but the dominant risk allele can be population dependent so a negative genotype is risk-reducing rather than exculpatory except where a near-complete negative predictive value has been validated, as for HLA-B*57:01 and abacavir. (**B**) The positive predictive gap: most individuals who carry a risk allele and are exposed to the drug never develop SCAR, so a positive HLA genotype is not sufficient and indicates increased rather than deterministic risk. In both directions the residual risk is accounted for by additional genetic variation, environmental and infectious cofactors, drug-related factors including dose and co-administered agents, and individual immune factors such as the T-cell receptor repertoire and prior heterologous immunity. (**C**) The number needed to test to prevent one case, which is the practical consequence of those two gaps. It is the reciprocal of the product of three independent inputs: the carriage frequency of the risk allele in the population being screened, the positive predictive value of the allele for the phenotype, and the prevalence of the phenotype among those exposed. Because all three vary by population, the same association can be highly cost-effective in one setting and impractical in another; co-trimoxazole spans a wide range for the additional reason that the relevant alleles differ by region

NNT, number needed to test (Fig. 3C); CPIC, Clinical Pharmacogenetics Implementation Consortium; DILI, drug-induced liver injury; NPV, negative predictive value; SJS/TEN, Stevens–Johnson syndrome/toxic epidermal necrolysis; DRESS, drug reaction with eosinophilia and systemic symptoms. Positive predictive value is below 10% for every association listed except abacavir and vancomycin, which is why a positive result is interpreted as increased risk rather than as a diagnosis. Geographic distribution of the SCAR-associated alleles is shown in Fig. 2. DILI associations are compiled from Nicoletti et al. and Krantz, Marks and Phillips [61, 62]; note that for allopurinol, carbamazepine and flucloxacillin the allele associated with liver injury may differ from, or coincide with, the allele associated with the cutaneous phenotype. Phenotypes overlap: DRESS frequently involves the liver, so the boundary between DRESS and DILI is not absolute, and several alleles listed here are associated with both a cutaneous and a hepatic phenotype - HLA-A*31:01 with carbamazepine, HLA-A*34:02 and HLA-B*58:01 with allopurinol, and HLA-B*57:01 with abacavir hypersensitivity and with flucloxacillin liver injury.

### Drug-induced Autoimmune Phenotypes

A smaller literature links drugs to phenotypes that are autoimmune rather than classically hypersensitivity in character. Here the associations are with class II alleles and the mechanism appears to be loss of tolerance to a self antigen rather than presentation of a drug-modified determinant. Bullous pemphigoid associated with dipeptidyl peptidase-4 inhibitors is best characterized: HLA-DQB1*03:01 was present in most Japanese patients with the non-inflammatory gliptin-associated phenotype and in a minority of gliptin-exposed controls, and variants at HLA-DQA1 have also been implicated [63, 64]. Drug-induced lupus, classically associated with hydralazine, procainamide, isoniazid and minocycline and more recently with TNF inhibitors, has been linked to HLA-DR4 alleles including HLA-DRB1*04:01, with HLA-DRB1*03:01 reported for sulfasalazine-associated disease; slow NAT2 acetylator status and complement C4 null alleles are additional modifiers, the acetylator association being a further example of a pharmacokinetic gene shaping an immunologically mediated phenotype [65]. Drug-associated ANCA vasculitis, which may be indistinguishable from granulomatosis with polyangiitis and is described with levamisole-adulterated cocaine, hydralazine, propylthiouracil and minocycline, occurs on the same class II background as idiopathic disease, in which HLA-DPB1*04:01 is strongly associated with proteinase 3–ANCA positivity and a proteinase 3 epitope binds HLA-DPB1*04:01 with high affinity and stimulates autoreactive T cells [66]. These associations have lower odds ratios and uneven replication, and none is currently actionable; their value is conceptual, showing that HLA restriction extends beyond cutaneous and hepatic phenotypes and that a drug can precipitate autoimmunity without ever functioning as an antigen itself.

### HLA is Necessary but not Sufficient

For most well-characterized associations, carriage of the primary identified risk allele may not be necessary or ​sufficient, even though the presence of an HLA association overall is necessary. Positive predictive values can range from less than 1% to 55%, so most carriers exposed to the drug never react (Fig. 3B), and reactions occur in patients who do not carry the allele that was tested, so a negative result does not identify everyone at risk (Fig. 3A). Non-carrier status should be read narrowly therefore if an attempted association and/or replication study has not been done in the population at hand. Through this review it denotes absence of the primary risk allele identified in the population in which the association was originally described, not absence of genetic risk. Some such patients carry a second, independently acting allele that the assay does not interrogate such as HLA-A*34:02 in allopurinol-induced SCAR. Because both the frequency of a given allele and the identity of the allele that carries the dominant risk differ between populations, the same negative result means different things depending on which allele was tested and in whom, and an association established in one population cannot be assumed to define the risk allele in another. Several variables interact to determine outcome. ERAP1 and ERAP2 variants alter peptide processing and modify abacavir risk even among HLA-B*57:01 carriers [27]. Metabolizing enzyme variation contributes, and CPIC advises interpreting HLA-B*15:02 alongside CYP2C9 status for phenytoin because CYP2C9*3 carriers clear the drug more slowly [67]. Heterologous immunity, in which T-cell repertoires shaped by prior HIV, EBV or CMV infection provide cross-reactive cells able to respond to drug-modified self-HLA, is a further mechanism [30]. KIR signaling and checkpoint control set effector activation thresholds. For diagnostic use, a negative result lowers the post-test probability of attribution only by the magnitude of the negative likelihood ratio and does not exclude a drug [1, 31]. In practice we communicate a positive genotype as evidence of increased rather than deterministic risk, and a negative genotype as risk-reducing but not exculpatory, except where a near-100% negative predictive value has been validated. Risk prediction will therefore need multifactorial models rather than single-allele testing.

### Beyond HLA and Immunogenomics

Not all heritable risk for SCAR sits within the MHC. Phenytoin-related SJS/TEN and DRESS are associated with reduced-function CYP2C9 variants, particularly CYP2C9*3, independent of HLA-B*15:02 and recognized in CPIC guidance recommending dose reduction in intermediate and poor metabolizers [67, 68]. Nevirapine separates the two contributions most clearly: slow-metabolizer CYP2B6 genotypes, principally 516G > T, raise plasma concentrations and are associated with hepatotoxicity and cutaneous reactions, while HLA carriage determines which phenotype develops, HLA-DRB1*01:01 with hepatitis in European-ancestry populations and HLA-C*04:01 with SJS/TEN in sub-Saharan Africa [69, 70]. Variation raising drug or metabolite exposure and variation determining whether that exposure is presented to T cells are complementary rather than competing explanations, and testing either alone accounts for less of the observed risk than testing both. The allergist and immunologist also encounters non-HLA pharmacogenetics unrelated to hypersensitivity, including TPMT and NUDT15 for thiopurine dosing, CYP2C19 for voriconazole, and glucose-6-phosphate dehydrogenase deficiency for dapsone.

## Genotype as an Adjunctive Diagnostic Test

The same drug–HLA association serves two clinical purposes. Prospectively it screens patients before prescribing, where positive and negative predictive value and the number needed to test determine utility [49]. Retrospectively it attributes a probable culprit among several co-administered drugs, where likelihood ratios and Bayesian pre- and post-test probabilities determine utility [71].

Patients with SCAR usually receive several potentially causative medications concurrently. Rechallenge is contraindicated in SJS/TEN and DRESS, and available in vivo and ex vivo tests have variable sensitivity, so attribution often remains unresolved. Where a validated association exists, HLA typing provides adjunctive evidence alongside the clinical phenotype, as with the prolonged latency of approximately 24–28 days and renal involvement typical of HLA-A*32:01-associated vancomycin DRESS [2, 5, 54]. ALDEN is an SJS/TEN-specific causality assessment tool. It scores exposure timing, drug notoriety, dechallenge, previous exposure, competing drugs and alternative causes, providing a clinical framework for interpreting genotype results [72]. The Naranjo algorithm is a general 10-item questionnaire that grades causality by probability and applies across drug reaction types but is not specific to cutaneous or immune-mediated reactions [73]. Genotype also helps distinguish immune-mediated hypersensitivity from a dose-dependent pharmacologic reaction mislabeled as drug allergy, supporting correction of an inaccurate label and dose adjustment rather than avoidance of an entire class, and informing whether skin testing, in vitro testing or graded challenge is the appropriate next step [1]. Table 2 sets out a six-step framework for applying these principles at the bedside. In our view, the most promising opportunity beyond established pre-prescription screening is the selective use of validated HLA associations to refine culprit attribution in multidrug SCAR.

**Table 2:** A practical framework for applying genotype at the bedside

Step	Action	What the genotype contributes	Caveat
1. Classify the reaction	Establish timing (immediate ≤ 6 h versus delayed > 6 h) and screen for SCAR red flags: mucosal or ocular involvement with skin detachment; fever, facial edema, eosinophilia and 2–8 week latency; sterile pustules with neutrophilia. Assess liver enzymes separately.	Nothing yet. Phenotype and chronology determine which associations are even relevant.	Phenotype is not pathognomonic of endotype; a delayed exanthem may be an evolving SCAR.
2. If no SCAR features	Proceed with phenotype-appropriate drug allergy evaluation: skin testing, in vitro testing or graded challenge as indicated.	No role. There is no validated genetic test for immediate reactions.	Do not order HLA typing to evaluate a low-risk immediate reaction.
3. If SCAR is suspected	Stop all non-essential suspected culprits, provide supportive and specialist care (dermatology, ophthalmology, intensive care), do not re-challenge, and list every potentially culpable medication with its exposure dates.	No role at this stage; treatment must not wait for a genotype.	Delay in withdrawing the culprit worsens outcome regardless of genotype.
4. Attribute causality	Obtain HLA typing for each co-suspect drug that has a validated association. Convert the pre-test probability of attribution to odds, apply the likelihood ratio, and convert back to a post-test probability.	Shifts probability between co-administered drugs; most useful when several plausible culprits were started together.	A positive result raises but does not establish attribution; a negative result does not exonerate a co-suspect drug.
5. Act and document	Record permanent avoidance of the most likely culprit in the EHR problem list. Re-evaluate co-suspect drugs with specialist input.	Supports a durable avoidance label where the genotype and phenotype agree.	Delabeling or continuation decisions should rest on phenotype-appropriate testing, not genotype alone.
6. Close the loop	Record actionable genotypes in the EHR so they are available prospectively, and apply them according to CPIC, regulatory and local prescribing guidance.	Converts a one-off diagnostic result into a lifelong prescribing safeguard, including for other drugs sharing the allele.	Requires decision support to be effective; a result buried in a scanned report changes nothing.

SCAR, severe cutaneous adverse reaction; EHR, electronic health record.

## Testing Methods: Genotyping and Functional Diagnostics

Implementation requires layered methods that differ in resolution, throughput and clinical use. Real-time PCR allele-specific assays are standard for clinically actionable alleles such as HLA-B*57:01, HLA-B*15:02 and HLA-B*58:01, offering rapid turnaround at low cost with minimal infrastructure. Next-generation sequencing provides full four-field typing across all HLA loci and is the research gold standard. HLA imputation from genome-wide single-nucleotide polymorphism arrays is less costly, but accuracy depends on the reference panel and is lower in ancestry groups underrepresented in genomic datasets [74, 75].

Functional testing identifies drug-reactive T cells in an individual patient and complements genotyping. Intradermal testing is useful in DRESS and AGEP but is not recommended in SJS/TEN given the risk of recall reactions [76]. Lymphocyte transformation testing and IFN-γ ELISpot measure drug-specific reactivity in peripheral blood mononuclear cells, with sensitivity dependent on timing relative to the acute phase, and blister fluid provides an enriched cell source that improves detection [77]. Biomarkers of inflammation and cellular injury such as granulysin and IL-15 may provide early diagnostic evidence in SJS/TEN. Because rechallenge cannot safely serve as a reference standard, diagnostics that avoid re-exposure remain a pressing need [78]. Machine-learning image classifiers have distinguished early SJS/TEN from its mimics with performance exceeding that of participating dermatologists, although external validation and integration with clinical data are required before routine use [79]. Panel-based testing combining HLA typing with metabolic enzyme variants becomes economically attractive when several actionable genes are screened together [80].

## Implementation: Guidelines, Cost-Effectiveness, and Equity

CPIC maintains 29 active guidelines covering 35 genes and over 160 drugs, with SCAR-relevant recommendations for abacavir and HLA-B*57:01, carbamazepine and HLA-B*15:02 and HLA-A*31:01, allopurinol and HLA-B*58:01, oxcarbazepine and HLA-B*15:02, and phenytoin with CYP2C9 and HLA-B*15:02 [51, 67]. The Dutch, Canadian and French pharmacogenetics networks publish overlapping but not always concordant guidance [81]. The U.S. Food and Drug Administration maintains an extensive and regularly updated table of therapeutic products with pharmacogenetic biomarker information in FDA-approved drug labeling [51, 82].

Adoption lags availability. Positive predictive value is below 10% for every established association other than abacavir and vancomycin so test-negative patients require counseling about residual risk and test-positive patients require an acceptable alternative agent [83]. Access, reimbursement and turnaround times that exceed the window for urgent prescribing are recurring obstacles, as is inconsistent integration of pharmacogenomic decision support into electronic health records [51, 80, 83]. The same gap exists outside HLA: metabolism panels are used in transplantation, oncology and psychiatry but have penetrated general internal medicine and primary care little, largely for want of education in translating genotype to phenotype [51, 80, 81]. An alternative to ancestry-based screening is supported by United States data showing that HLA-B*15:02 carriage in non-Asian patients still carries clinical relevance [84].

The value of prospective screening depends on the number needed to test, a function of allele carriage frequency, positive predictive value and phenotype prevalence (Fig. 1C). Reported values span three orders of magnitude, from approximately 13 for abacavir and HLA-B*57:01 to approximately > 2000 for carbamazepine and HLA-A*31:01 and > 10,000 for HLA-B*57:01 and flucloxacillin DILI (Table 2). Screening is most favorable where the risk allele is prevalent, the reaction severe and acceptable alternatives available, and may be economically unfavorable where allele frequency is low [85]. Policy reflects this. Taiwan has mandated HLA-B*15:02 screening before carbamazepine since 2010, after which SJS/TEN fell to near zero in screened groups [86]. In Taiwan HLA-B*58:01 screening is recommended before allopurinol prescription, with prospective implementation studies showing a marked decline in allopurinol-induced SCAR [87]. Countries with central regulatory systems and integrated electronic prescribing have been the most successful in adopting pharmacogenetic screening [88].

The allele-centered model depends on populations that have been studied. The carbamazepine and allopurinol screening studies conducted in Taiwan enrolled participants of Han Chinese descent. In both studies, participants reported the ethnic origins of their parents and grandparents to establish eligibility [86, 87]. HLA screening based on perceived race or ethnicity is generally not recommended, as these socially defined categories are imperfect proxies for genetic ancestry and HLA-associated risk. The distribution of HLA risk alleles varies across geographic regions and populations reflecting patterns of genetic ancestry, migration and admixture. A more comprehensive understanding of the geographic and ancestral distribution of HLA risk alleles is therefore essential to inform equitable screening strategies, particularly in populations with substantial genetic admixture. Such knowledge may improve the identification of individuals at risk and help optimize the negative predictive value of HLA screening across heterogenous populations. Many HLA alleles remain poorly characterized in African, Indigenous American and South Asian populations, so an association established in one ancestry group may not translate to another, and recommendations based solely on ancestry stratification risk reinforcing inequities rather than correcting them [89]. Increasingly, shared peptide-binding specificities, reflecting the structural chemistry of the peptide-binding cleft and particularly key anchor-binding pockets such as the F pocket, may provide a more transferable framework for risk than individual HLA alleles. This approach could extend prediction to structurally related but understudied alleles across diverse populations, enabling a more equitable basis for implementation [78].

## Conclusions

Genetic risk for predictable pharmacologic and on-target adverse drug reactions sits primarily in metabolism and transporter genes, whereas immune-mediated risk associated with delayed drug reactions sits in the MHC. This dichotomy, reinforced by the EAACI phenotype, endotype and drug-characteristic framework, is what makes the HLA signal interpretable and clinically actionable [1, 2, 4, 5].

HLA pharmacogenetics now serves two distinct clinical roles: pre-prescription screening and post-reaction attribution among co-administered drugs [49, 51, 54, 71]. A small set of drug–HLA associations is genuinely actionable today. Genotype should be used as a risk modifier and diagnostic adjunct rather than a stand-alone test, interpreted against incomplete positive predictive value, residual risk after a negative result and ancestry-dependent allele frequencies [1, 31]. Priorities for the next decade include developing and validating multifactorial risk models that integrate HLA with additional genetic and biological modifiers, including ERAP and KIR variation, drug-metabolizing pathways, and other immune regulatory factors. Although several such modifiers have been associated with SCAR risk, their incremental predictive value beyond established HLA associations remains uncertain. Equally important is expanding genomic studies in historically underrepresented populations, to broadly represent global risk and to identify population-specific and shared determinants of risk. Both would strengthen the evidence base and support equitable access to care [85, 89]. Continued international cooperation linking clinical phenotyping, genomic discovery and policy will underpin next-generation SCAR prevention [2, 5, 51, 90].

## Key References

- Hung SI, Mockenhaupt M, Blumenthal KG, et al. Severe cutaneous adverse reactions. Nat Rev Dis Primers. 2024;10:30.
  - ○This article synthesizes the clinical manifestation, epidemiology, pathophysiology and genetics of SCARs. It serves as an updated reference for SCAR diagnosis, mechanism, and management.
- Torres MJ, Mayorga C, Phillips EJ, et al. Nomenclature on immune-mediated drug reactions: An EAACI position paper. Allergy. 2026. doi:10.1111/all.70480.
  - ○This position paper establishes the terminology used throughout this review, separating drug allergy (drug-antigen-driven reactions, diagnosed by tests detecting sensitization) from drug hypersensitivity (drug-directly-driven reactions). It also defines the diagnostic gap that pharmacogenetics is positioned to fill, since confirmatory tests for drug-directly-driven reactions are largely unavailable and phenotype alone cannot establish endotype.
- Gibson A, Deshpande P, Campbell CN, et al. Updates on the immunopathology and genomics of severe cutaneous adverse drug reactions. J Allergy Clin Immunol. 2023;151(2):289–300.
  - ○This review updates our understanding of SCAR mechanisms by combining new insights on HLA immunology. It highlights how HLA-restricted-T-cell activation plays a key role in SCAR manifestation and identifies significant gaps in implementing pharmacogenomics clinically.
- Gibson A, Ram R, Gangula R, et al. Multiomic single-cell sequencing defines tissue-specific responses in Stevens-Johnson syndrome and toxic epidermal necrolysis. Nat Commun. 2024;15:8722.
  - ○This study used single-cell transcriptomic, T-cell receptor, and surface proteomic profiling of SJS/TEN skin and blister fluid to reveal tissue-resident clonally expanded CD8⁺ T cells and define candidate biomarker and therapeutic axes including JAK-STAT and TNF signaling. These tissue-resident data complement bulk proteomic findings and support JAK inhibition as a mechanistically anchored therapeutic candidate, illustrating the utility of profiling the target organ rather than blood alone.
- Nordmann TM, Anderton H, Hasegawa A, et al. Spatial proteomics identifies JAKi as treatment for a lethal skin disease. Nature. 2024;635(8040):1001–1009.
  - ○Using deep visual proteomics to visualize toxic epidermal necrolysis biopsy samples, this breakthrough study found hyperactivation of JAK/STAT signaling as a key therapeutic target and showed clinical responses to JAK inhibitors in seven TEN patients. It is the first direct translation from spatial-omics to a clinical intervention for SCAR management.
- Caudle KE, Whirl-Carrillo M, Relling MV, et al. Advancing clinical pharmacogenomics worldwide through the Clinical Pharmacogenetics Implementation Consortium (CPIC). Clin Pharmacol Ther. 2025;118(6):1512–1522.
  - ○This CPIC update describes the 28 active pharmacogenomic guidelines published since 2017 that include recommendations for 34 genes and 164 medications as well as quantification of the CPIC's global utilization and its incorporation into electronic decision support systems. The article provides a view of the current state of translating SCAR pharmacogenetics into practice.
- Campbell CN, Krantz MS, Yu A, Phillips EJ; SJS/TEN Survivor Study Collaborators. HLA-B*58:01 and risk of allopurinol-induced severe cutaneous adverse reactions in the US. JAMA Dermatol. 2025;161(12):1258–1263.
  - ○This study identified HLA-A*34:02 as a risk allele for allopurinol-induced SCAR, providing an explanation for patients testing negative for the well-established HLA-B*58:01. It emphasizes the idea that single-allele screening may fail to identify at-risk patients in diverse populations.
- Omran S, Gan SH, Teoh SL. Pharmacogenomics in drug therapy: global regulatory guidelines for managing high-risk drug reactions. Eur J Hum Genet. 2026;34(1):27-36.
  - ○This review compares how Asian, European and North American countries regulate pharmacogenomics when prescribing a drug that has a high risk of adverse reaction such as carbamazepine. It documents significant differences in screening mandates for specific genetic variations. Additionally, it describes progress towards the development of integrated national frameworks.

## Data Availability

No datasets were generated or analysed during the current study.
